# Research progress on aero-optical effects of hypersonic optical window with film cooling

**DOI:** 10.1038/s41377-024-01596-x

**Published:** 2024-11-18

**Authors:** Shihe Yi, Haolin Ding, Suyiming Luo, Xiaobin Sun, Zihao Xia

**Affiliations:** College of Aerospace Science and Engineering, National University of Defense Technology (NUDT), Changsha, Hunan 410073 China

**Keywords:** Atmospheric optics, Fibre optics and optical communications

## Abstract

In recent years, the demand for optical imaging and detection in hypersonic aircraft has been on the rise. The high-temperature and high-pressure compressed flow field near airborne optoelectronic devices creates significant interference with light transmission, known as hypersonic aero-optical effects. This effect has emerged as a key technological challenge, limiting hypersonic optical imaging and detection capabilities. This article focuses on introducing the thermal effects and optical transmission effects of hypersonic aero-optical effects, as along with corresponding suppression techniques. In addition, this article critically reviews and succinctly summarizes the advancements made in hypersonic aero-optical effects testing technology, while also delineating avenues for future research needs in this field. In conclusion, there is an urgent call for further exploration into the study of aero-optical effects under conditions characterized by high Mach, high enthalpy, and high Reynolds number in the future.

## Abstract (web summary)

In response to the challenges of obscuring phenomenon faced by high-speed aircraft, this article focuses on the mechanism, testing, and suppression technology research progress of hypersonic aero-optical effects.

## Graphical abstract

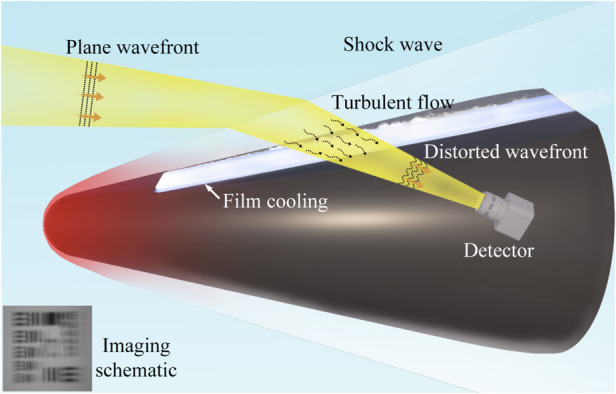

## Introduction

**Fig. 1:**
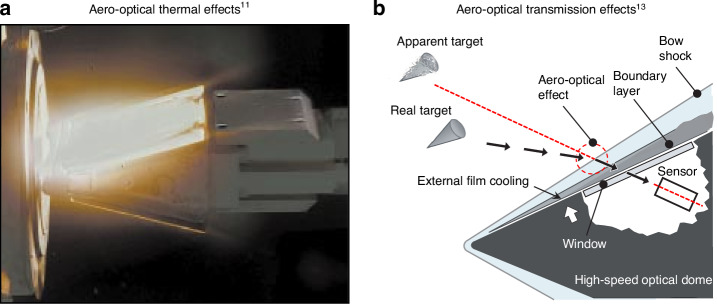
Aero-optical effects of hypersonic optical window. **a** Aero-optical thermal effects test in wind tunnel. **b** Schematic of aero-optical transmission effects

When an aircraft flies at hypersonic speeds (Mach number >5), the increased temperature and pressure flow structure near the optical window obstruct the detection of the forward targets by infrared detectors^1–4^. This phenomenon, known as hypersonic aero-optical effects, encompasses both aero-optical thermal effects and aero-optical transmission effects. Hypersonic aero-optical effects present challenges to the achievement of effective infrared imaging and detection in hypersonic conditions^5,6^. The critical issues that require attention are outlined as follows:

- Aero-optical thermal effects: in hypersonic conditions, the optical window of an aircraft is subjected to severe aerodynamic heating, leading to temperatures exceeding 2000 K. This results in the optical window and the surrounding gas to experience significant thermal loads, causing the gas radiation, window radiation and alterations in the physical properties of the window, overwhelming the infrared signals from the targets at further distances^3,7^. As a result, the infrared detectors become saturated and are incapable of detecting the targets^8–10^, leading to a visual obstruction phenomenon that is specific to hypersonic aero-optical effects. The ground Arc wind tunnel experiment shown in Fig. 1a involves a wedge-shaped test model emitting light as a result of high-enthalpy flow heating^11^.
- Aero-optical transmission effects: the intricate and highly compressed turbulent flow field surrounding a hypersonic optical window can introduce interference to the transmission of target light, resulting in blurred, jittery, and shifted target images^12^. This phenomenon, known as the aero-optical transmission effect^13,14^, hinders the effective imaging of the target by the guidance head, as illustrated in Fig. 1b.
- Hypersonic aero-optical wavefront measurement: during ground testing of hypersonic optical windows to evaluate the aero-optical transmission effect, traditional optical wavefront testing techniques face challenges due to low spatial and temporal resolution^15,16^. Moreover, the integration of factors such as jet shear layers in wind tunnel test setups introduces interference to the optical pathway^17^. As a result, these limitations make the wavefront wind tunnel tests of hypersonic optical window “imprecise”, posing challenges to effectively support studies on aero-optical effects.

The challenges mentioned above have imposed a speed limitation on target optical imaging detection for hypersonic aircraft. Thus, achieving optical imaging detection of ground, sea, and aerial targets at higher speeds is currently unfeasible. This has become a critical technological challenge that requires immediate attention to achieve optical imaging detection under hypersonic conditions^14,18,19^.

Over the years, in response to the challenges encountered in the development of optical windows for aircraft under hypersonic conditions, including hypersonic aero-optical visual obstruction phenomena, transmission effects, and wavefront measurements, this paper focuses on introducing the visual obstruction phenomena and optical transmission effects in hypersonic aero-optical effects, along with their corresponding suppression techniques. This paper provides a comprehensive review and summary of research advancements in testing techniques for hypersonic aero-optical effects, as well as future requirements. Finally, it emphasizes the urgent need for further exploration of aero-optical effects under conditions characterized by high Mach numbers, high enthalpy, and high Reynolds numbers.

## Aero-optical thermal effects of hypersonic optical window and its suppression

The essence of infrared imaging detection is to detect the difference in radiation energy levels between the target and the background. Presently, the noise equivalent temperature difference of supersonic infrared detection systems generally falls below 100 mK. Under hypersonic conditions, the temperatures of optical windows, shockwaves, and other near-field high-temperature radiation sources can reach hundreds or even thousands of degrees Celsius, which is much higher than the temperature of the target background, as shown in Fig. 1a. The thermal radiation interference originating from these high-temperature sources reduces the signal-to-noise ratio of the system^7^. In severe cases, this interference can even obscure the target signal or saturate the detector. At the same time, the heating window can also cause changes in the internal refractive index of the material and even result in a certain degree of window deformation, thereby influencing the direction of signal transmission. These phenomena are collectively referred to as aero-optical thermal effects.

### Gas radiation effects of hypersonic optical window

**Fig. 2:**
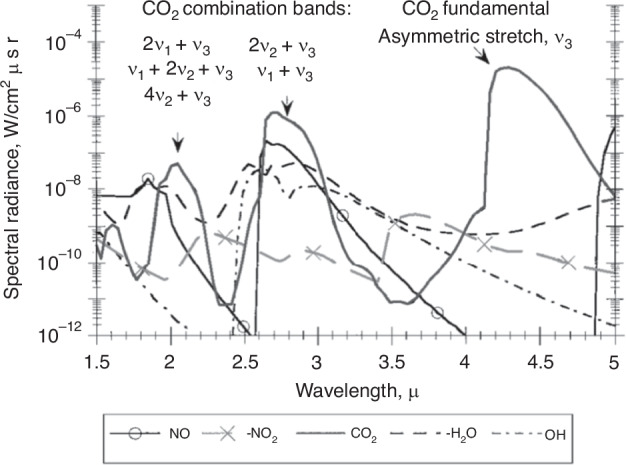
Calculated spectra at 40 km altitude, 3.5 km s^−1.^^21^

During hypersonic flight, the gas undergoes strong compression, resulting in the generation of shockwaves near the optical window. After the shockwaves, the temperature and density of the gas increase, causing gas radiation due to molecular rotation, vibration, and electron energy level transitions. In 1992, Trolier et al.^20^ conducted a study on the flow field radiation characteristics and transmittance of a typical endo-atmospheric interceptor. Clearly, the main sources of gas radiation are CO_2_ and NO generated by air ionization, with radiation spectra concentrated at 3.44 μm and 6.2 μm. As shown in Fig. 2, Levin et al.^21^ also reached a similar conclusion. At a velocity of 3.5 km s^−1^, radiation from the ambient CO_2_ heated in the shock layer was identified as the major contribution between the altitudes of 30 and 60 km. Further research by Wu et al.^22^ found that under typical hypersonic flight conditions (30~50 km, 5 km s^−1^), the infrared radiation of CO_2_ on the stagnation line in a hypersonic flow field without ablation is significantly lower than that of the aircraft body. In the case of ablation, the infrared radiation of CO_2_ in the shock layer cannot be ignored. In 2017, Gao et al.^23^ analyzed the characteristics and patterns of infrared spectral radiation in the flow field near an optical window as a function of flight parameters. When the flight Mach number remains constant, the variation of spectral radiation in the window flow field along the flight altitude is mainly dominated by the number density distribution of gas molecules in the flow field. At a certain flight altitude, the variation of spectral radiation in the window flow field with flight Mach number is primarily determined by the temperature of the flow field. With an increase in flight Mach number, the spectral radiation effect of NO molecules in the flow field is enhanced.

Regarding the main sources and concentrated spectral characteristics of gas infrared radiation, Trolier et al.^20^ suggested injecting radiation-suppressing substances into the thermal shock layer and selecting appropriate infrared detectors to mitigate the impact of gas radiation within the shock layer on imaging. Combining the performance evaluation method of an infrared detection system under aerodynamic thermal radiation environment, Fei et al.^24^ proposed the utilization of spectral filtering design to reduce the radiation effect of gas within the shock wave layer, in order to improve the signal-to-noise ratio of infrared imaging systems for target detection under high-speed flight conditions.

**Fig. 3:**
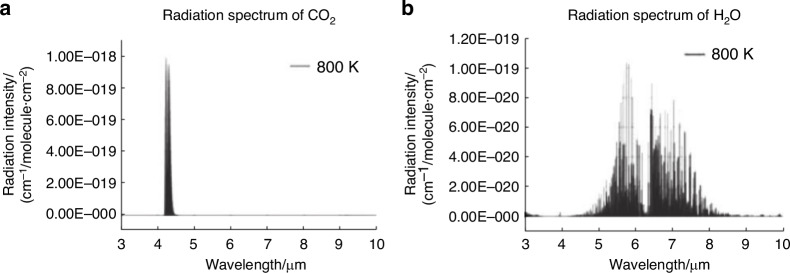
Radiation spectrum of CO_2_ and H_2_O calculated by HITRAN^26^. **a** Radiation spectrum of CO_2_ at 800 K. **b** Radiation spectrum of H_2_O at 800K

Overall, based on the research findings of numerous scholars, it is generally believed that the influence of gas thermal radiation on imaging signal-to-noise ratio is perceived to be relatively minor^20–25^. The radiation spectrum covers a range from ultraviolet to long-wave infrared wavelengths. N_2_ and O_2_, which constitute a substantial portion of the air, possess fixed dipole moments and do not exhibit infrared radiation characteristics. On the contrary, as shown in Fig. 3, CO_2_ and H_2_O, which constitute a minor portion of the air, possess non-fixed dipole moments and exhibit strong infrared radiation characteristics^26^. Exactly due to this reason, the intensity of gas radiation is significantly lower than the thermal radiation intensity of the high-temperature optical window itself. Therefore, in practical engineering problems, the primary emphasis lies in effectively reducing the temperature of the optical window.

### Radiation and physical properties variation of heating window

**Fig. 4:**
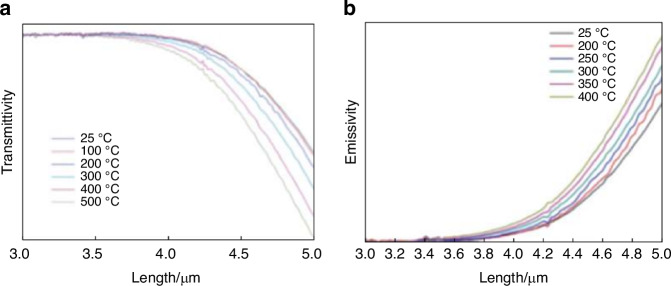
Spectral transmittivity and emissivity of sapphire window^26^. **a** Spectral transmittivity of sapphire window at different temperatures and wavelengths. **b** Spectral emissivity of sapphire window at different temperatures and wavelengths

During hypersonic flight, aerodynamic heating causes a rapid increase in the temperature of the optical window, resulting in significant thermal stress. It also leads to a decrease in the transmittance of the window and an increase in its own radiation^7^. As shown in Fig. 4, with the temperature increases, the transmittance of the sapphire window decreases significantly, while its own radiation increases significantly^19^. The self-radiation of the optical window enhances the background brightness of the infrared image, while reducing the transmittance and increasing the loss of the target signal.

**Fig. 5:**
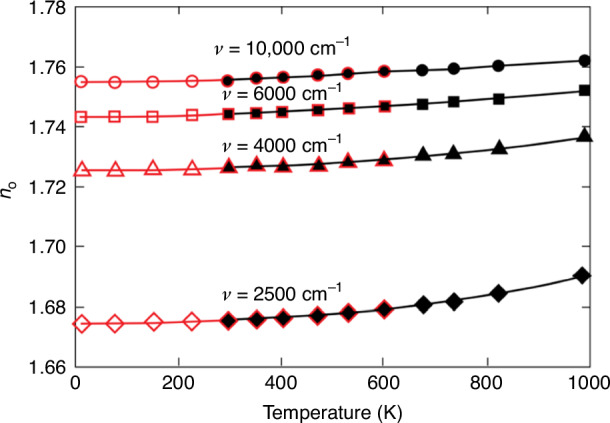
Experimental estimates of the refractive index of o-ray sapphire *n*_0_ from NIST (red) and APL (black)^3^

Duncan et al.^3^ conducted an experimental and theoretical evaluation of mechanical and optical effects in nonuniformly heated IR windows. Figure 5 shows the experimental estimates of the refractive index (*n*_0_) as a function of temperature below and above room temperature at wavelengths of 1 μm, 1.7 μm, 2.5 μm, and 4 μm. As observed in this study, the results match well within the temperature range where NIST (National Institute of Standards and Technology) and APL (Johns Hopkins University Applied Physics Laboratory) experiments overlap. The thermal variation throughout the thickness of the dome results in a refractive index gradient that causes curvature of the ray trajectories. For typical conformal windows, Fan et al.^27^ found that image degradation is mainly caused by the refractive index distribution of the conformal dome rather than its deformation.

### Suppression of aero-optical thermal effects

**Fig. 6:**
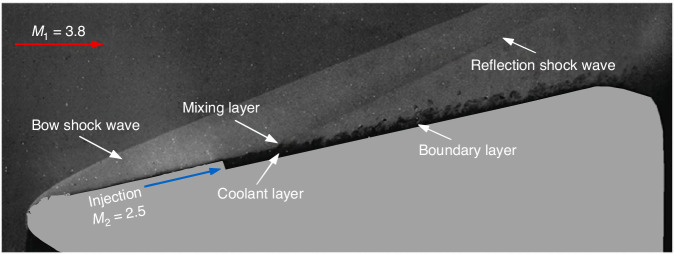
Flow structure near the optical window of hypersonic optical window with supersonic film cooling^5^

In order to reduce the impact of aerodynamic heating on the optical windows and eliminate the visual obscuration phenomenon caused by hypersonic aerodynamic heating, an effective approach is to achieve reliable cooling for the optical windows. Therefore, numerous scholars have carried out extensive explorations and research, proposing various window cooling methods, including internal channel window cooling, refrigerated mosaic windows, external discrete slot film cooling, oblique film cooling, and tangential film cooling, with the aim of providing insulation from high-temperature mainstream^5,28–30^. Numerous experiments and calculations have demonstrated that tangential film cooling not only achieves reliable cooling for side windows but also offers a simpler structure in comparison to internal channel cooling methods. It does not affect the light transmission efficiency^31^ and causes relatively little interference with the transmission of light^30,32^. In the following text, tangential film cooling is abbreviated as film cooling. Figure 6 is the typical flow visualization results of supersonic film cooling for hypersonic optical window^5^.

**Fig. 7:**
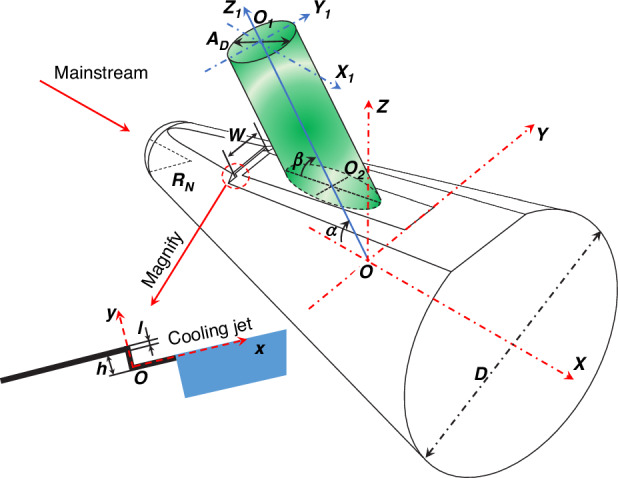
Schematic diagram of geometric parameters and imaging beam parameters of optical window with supersonic film cooling^33^

When designing a hypersonic optical window with film cooling, it is necessary to consider the combined impact of multiple design elements, including imaging guidance systems, aero-optical effects, windows thermal protection, and aerodynamic drag. As shown in Fig. 7, in line with these design requirements, a fundamental model for hypersonic optical windows with supersonic film cooling has been established^33^. In general, the design of hypersonic optical windows involves a combination of factors, including head heat flux index, imaging system index, cooling performance index, aerodynamic performance index, volume ratio index, and the degree of aero-optical effects^5^. In the 1980s and 1990s, Majeski et al.^28,34,35^ conducted a series of tests in multiple wind tunnels to develop a predictive model for film cooling effectiveness (*η*) adapted to planar optical windows.

1 $$\eta =a{({S}^{\ast })}^{b}$$

In Eq. (1), ${S}^{\ast }=(\frac{x}{h\lambda }){(\frac{R{e}_{{\rm{c}}}{\mu }_{{\rm{c}}}}{{\mu }_{\infty }})}^{-0.25}{(\frac{{\rho }_{{\rm{c}}}}{{\rho }_{\infty }})}^{0.4}{(\frac{{\mu }_{\infty }}{{\mu }_{{\rm{c}}}})}^{0.75}{(1+\frac{\gamma -1}{2}{{M}_{{\rm{c}}}}^{2})}^{-0.5}$; *a* and *b* are fitting constant; *Re*_c_ is the jet outlet Reynolds number, and$R{e}_{{\rm{c}}}={\rho }_{{\rm{c}}}{u}_{{\rm{c}}}h/{\mu }_{{\rm{c}}}$; *μ*_c_, *ρ*_c_ and *μ*_∞_, *ρ*_∞_ are the dynamic viscosity coefficient and density of the jet and mainstream, respectively; *γ* is the specific heat ratio of jet gas; *M*_c_ is the jet Mach number.

**Fig. 8:**
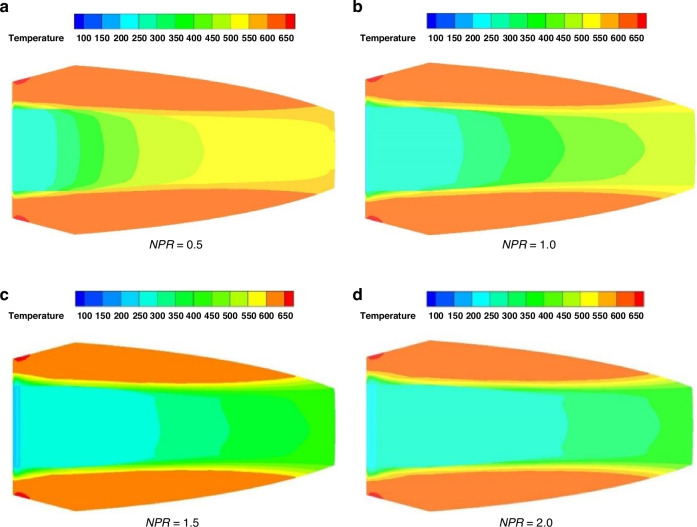
Cloud image of surface temperature distribution of optical window under different *NPR*^36^. **a** Supersonic cooling fim operating at *NPR* = 0.5. **b** *NPR* = 1.0. **c** *NPR* = 1.5. **d** *NPR* = 2.0

The introduction of the cooling film requires the aircraft to carry a significant quantity of coolant. Additionally, the limited space within the guidance head further constrains the application of film cooling. Therefore, it is necessary to optimize the design of film cooling to improve the utilization of coolant, by employing a lower mass flow rate to achieve a greater cooling range. Based on experimental and numerical simulation methods at a mainstream Mach number of 7.1, the supersonic film cooling law for a typical hypersonic optical window was studied for nozzle pressure ratio (*NPR* = jet outlet static pressure / nearby mainstream static pressure) ranging from 0 to 2.3. As shown in Fig. 8, it was found that with increasing *NPR*, the film cooling effectiveness gradually improved, corresponding to an expansion of the effective cooling zone^36^.

In order to better evaluate the cooling performance of the film, Ding et al.^37^ proposed to obtain the “unit cooling length” by dividing the effective cooling length of the supersonic film by the mass flow rate of the coolant consumed by the supersonic film.

2 $${C}_{\dot{m}}=\frac{{x}_{{\rm{cl}}}}{\dot{m}}$$

Where ${x}_{{\rm{cl}}}$ is the effective cooling length of the supersonic film; $\dot{m}$ is the mass flow rate of the coolant, defined as $\dot{m}={\rho }_{{\rm{c}}}{u}_{{\rm{c}}}A$, *ρ*_c_ and *u*_c_ are the density and velocity of the film, and *A* is the area of the jet outlet.

**Fig. 9:**
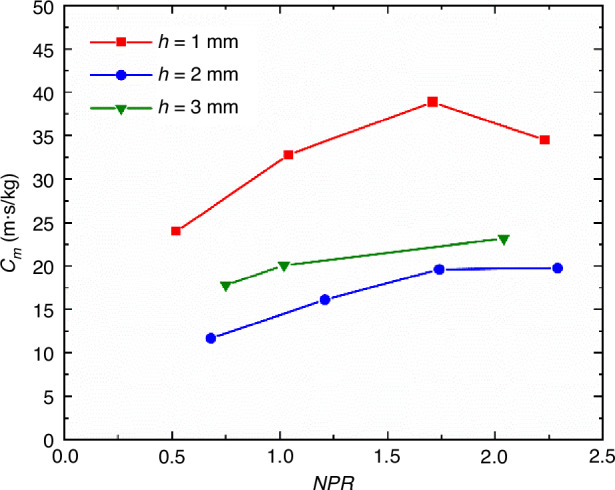
The “unit cooling length” at different *NPR*^37^

**Fig. 10:**
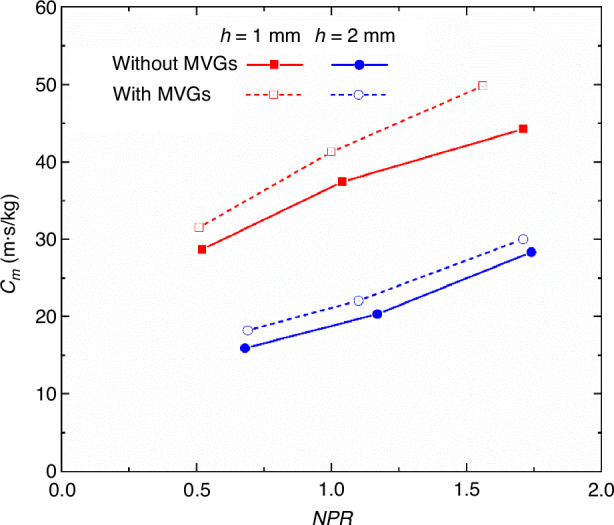
The “unit cooling length” with/without MVGs at different *NPR*^38^

As shown in Fig. 9, with the increase of *NPR*, the effective cooling length corresponding to the unit mass flow rate of the coolant first increases and then decreases or becomes stable, and there is an optimal solution. Furthermore, at the same coolant mass flow rate, reducing the jet outlet height and increasing the jet Mach number can enhance the supersonic film cooling performance. Meanwhile, the impact of the microvortex generators (MVGs) array installed upstream of the film outlet on the supersonic film cooling performance was studied. Ding et al.^38^ found that following the control of mainstream by the MVGs array, the surface heat flux on the optical window decreased by over 30%, thereby significantly improving the effective cooling length corresponding to the unit mass flow rate of the coolant, as shown in Fig. 10.

In general, as hypersonic aircraft continue to advance towards higher speeds, further research is still required to better enhance the suppression of optical dome obscuration phenomena under hypersonic, high enthalpy, and high Reynolds number conditions. Firstly, to meet the multi-parameter modeling requirements of the film cooling prediction model for the hypersonic optical dome, it is essential to enhance the model by incorporating the effects of jet and mainstream parameters on film cooling performance. This will offer theoretical support for the optimization design of film cooling for hypersonic optical windows. Secondly, it is urgent to conduct high-precision numerical simulations to reveal the interaction mechanisms between the hypersonic mainstream and the supersonic film. These simulation results will aid in developing a flow zoning model for supersonic film cooling of hypersonic optical windows. Finally, there is a need for comprehensive research on the film cooling and the aero-optical effects of the hypersonic optical window. By integrating aerodynamics, thermodynamics, and optics, the coupling design model of film cooling performance and optical performance suitable for a hypersonic optical window is constructed to achieve optimal film cooling and smaller optical distortion.

## Aero-optical transmission effects of hypersonic optical window and its suppression

### Influence of turbulent vortex structure characteristics on aero-optical effects

The theory of hypersonic aero-optical transmission effects serves as the theoretical basis for the suppression of aero-optical distortion of hypersonic optical windows. Research indicates that the primary underlying mechanism behind the aero-optical transmission effects of hypersonic optical windows is the variation in density (refractive index) in the flow field near the optical window, and turbulent vortices are the main carriers of these density variations^39,40^. In 1956, Stine and Winovich^41^ initiated a study about the energy scattering distribution of light traversing a turbulent boundary layer and established the model of the boundary layer’s velocity on light intensity. Subsequently, researchers such as Steinmetz^42^, Havener^43^, Sutton^44^, Wyckham^45^, Gordeyev^46,47^, and others have further expanded and refined the aero-optical distortion prediction model relevant to the turbulent boundary layers^48^.

For subsonic, supersonic, and high-supersonic flow, the radiation effect of the gas itself can be considered negligible. Therefore, the aero-optical transmission effects can be abbreviated as the aero-optical effects. The predominant aero-optical effect after transmission through the turbulence region is a phase distortion of the optical wavefront, quantifiable by the optical path length (*OPL*). In practice, the relative variance in the *OPL* over the aperture serves as a more pertinent representation of wavefront distortions. It is termed the optical path difference (*OPD*). The spatial root mean square of *OPD*(*x*, *y*, *t*), denoted as *OPD*_rms_(*t*), is commonly employed to quantify the intensity of aero-optical effects. To facilitate analysis and mitigation of distortions, researchers often decompose the time-dependent *OPD* into a time-averaged spatial component, called the steady-lensing term, denoted as *OPD*_steady_(*x*, *y*), and an unsteady component^15^. The unsteady component can be further divided into a spatially linear component, called unsteady tilt or beam jitter, and the remaining components, typically denoted as high-order distortions.

3 $${OPD}(x,y,t)={{OPD}}_{{\rm{steady}}}(x,y)+[A(t)x+B(t)y]+{{OPD}}_{{\rm{high}}-{\rm{order}}}(x,y,t)$$

In Eq. (3), the steady-lensing term, *OPD*_steady_(*x*, *y*), solely depends on the time-averaged density field and imposes a steady distortion such as a defocus or coma. The tilt or jitter, represented by the second term on the right-hand side, does not change the spatial distribution of the outgoing beam but simply redirects it in directions defined by functions *A*(*t*) and *B*(*t*). The high-order term, *OPD*_high-order_, results in changes to the beam’s shape and intensity distribution.

**Fig. 11:**
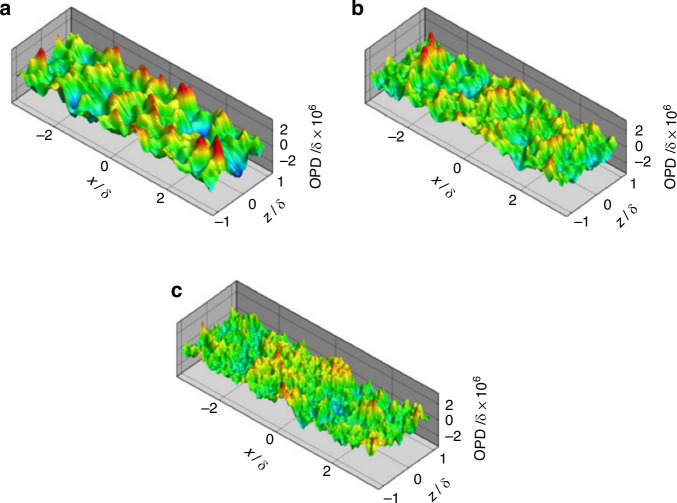
Instantaneous *OPD* at a time instant for different Reynolds numbers. **a** Instantaneous *OPD* of turbulent boundary layer for *Re*_*θ*_ = 875. **b** *Re*_*θ*_ = 1770. **c** *Re*_*θ*_ = 3550^49^

**Fig. 12:**
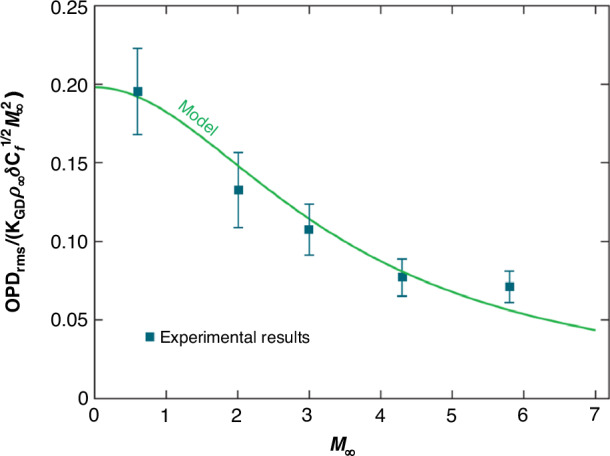
A comparison of the ND model with experimental data for supersonic and high-supersonic turbulent boundary layers^51^

For subsonic turbulent boundary layers, Wang and Wang^49^ conducted compressible large-eddy simulation (LES) to investigate the aero-optical distortions induced by Mach 0.5 flat-plate turbulent boundary layer at different Reynolds numbers, as depicted in Fig. 11. The Reynolds numbers considered were *Re*_*θ*_ = 875, 1770, and 3550 based on the momentum thickness. Concurrently, in-depth discussions were conducted on fundamental issues related to aero-optics^15,50^. Gordeyev et al.^46^ provided a thorough characterization of the aero-optical effects induced by a subsonic, compressible, turbulent boundary layer. Furthermore, they constructed a highly effective prediction model (ND model) for subsonic, supersonic, and high-supersonic turbulent boundary layer aero-optical effects^51^, as shown in Fig. 12.

**Fig. 13:**
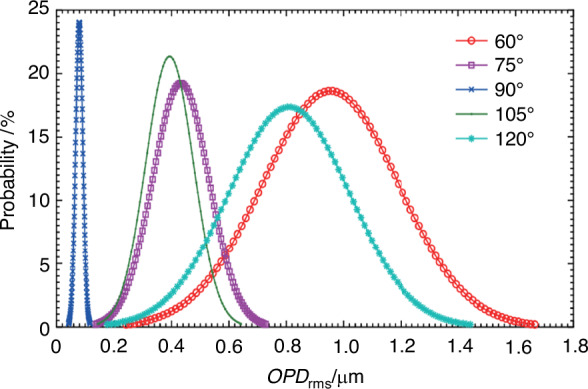
*OPD*_rms_ probability distribution of supersonic turbulent boundary layer at different light incident angles^52^

For supersonic (Mach 3.0) turbulent boundary layers, Ding et al.^52^ clarified the influence of the anisotropy of turbulent vortex structures on light transmission by using the generalized aero-optical linking equation and the spatial two-point correlation method based on density fluctuations. The aero-optical transmission effects are mainly related to the distance of light propagation and the correlation of density fluctuations along the light propagation path within the flow field. Furthermore, the causes behind the increase in the amplitude and non-uniformity of aero-optical transmission effects induced by oblique light incidence are explained, as shown in Fig. 13. The *OPD* serves as a crucial indicator for characterizing the aero-optical transmission effects. The spatial root mean square of *OPD* (*OPD*_rms_) within the optical window is used to represent the intensity of the aero-optical transmission effects.

**Fig. 14:**
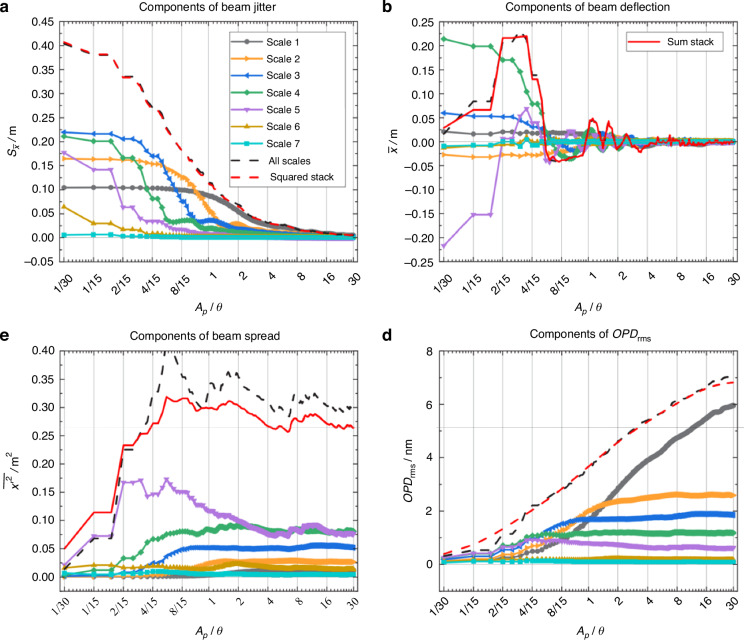
The influence of turbulent scales on aero-optical distortion under different optical window sizes^53^. **a** Components of beam jitter induced by different turbulent scales. **b** Components of beam deflection induced by different turbulent scales. **c** Components of beam spread induced by different turbulent scales. **d** Components of *OPD*_rms_ induced by different turbulent scales

Furthermore, Luo et al.^53^ identified turbulent structures of varying characteristic scales through the wavelet multi-scale decomposition. As shown in Fig. 14, they found that there is a “coupling effect” between the optical aperture and the characteristic scale of turbulence structure on the aero-optical transmission effects. As the size of the optical window increases, the proportion of turbulent structures larger than the optical window decreases, which can suppress the jitter and displacement of the light beam. This conclusion is consistent with the research results of Wang et al.^49^ on subsonic boundary layers. More details about the aperture effect can be found in ref. ^54^.

**Fig. 15:**
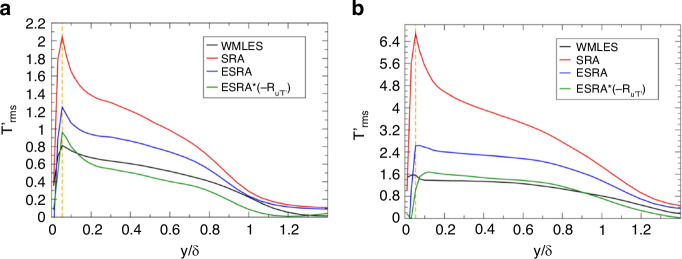
Aperture-averaged temperature fluctuations obtained directly from WMLES and from SRA^55^. **a** Mach 8 turbulent boundary layer. **b** Mach 14 turbulent boundary layer

In a hypersonic environment, the high kinetic energy of the oncoming flow results in thermal excitation of the flow molecules, leading to dissociation^4^. For hypersonic turbulent boundary layers, Gomez et al.^55^ used a WMLES to compute a Mach 14 boundary layer flow over a flat-plate for the conditions of the Arnold Engineering Development Complex Hypervelocity Tunnel 9. The *OPD*_rms_ obtained from the present WMLES below a prediction obtained from a semi-analytical relationship by Notre Dame University. This conclusion is consistent with Castillo et al.^56,57^ and Miller et al.^16^ on Mach 8 and 14 turbulent boundary layers. Gomez et al.^55^ further elucidated the reasons for the above phenomenon. As Mach number increases, the pressure fluctuations escalate, and the strong Reynolds analogy (SRA) over-predicts the temperature fluctuations, as shown in Fig. 15.

**Fig. 16:**
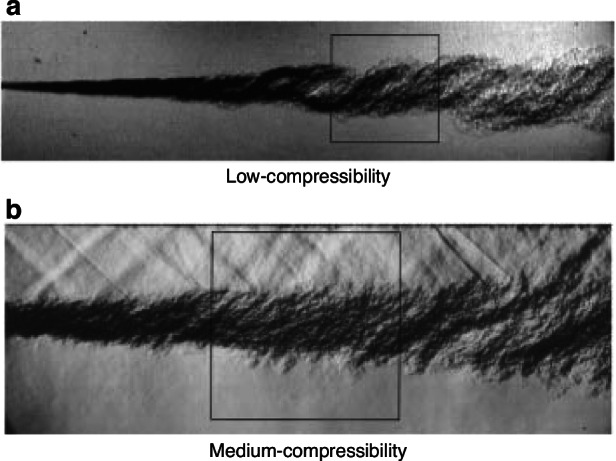
Schlieren image of shear layers^63^. **a** Shear layer with low-compressibility. **b** Shear layer with medium-compressibility

The aero-optical distortions caused by supersonic shear layers over an optical window are crucial to the performance of hypersonic vehicles^48,58^. Consequently, the aero-optical effects induced by supersonic shear layers need particular attention in both fundamental research and engineering endeavors^48^. Jumper et al.^59–62^ conducted extensive studies on the weakly compressible turbulent shear layer and proposed multiple beneficial aero-optical effect prediction models systematically. Dimotakis et al.^63^ reported on the structure of the scalar index-of-refraction field generated by turbulent, gas-phase, low-compressibility (Fig. 16a) and medium-compressibility (Fig. 16b) shear layers, and on associated beam-propagation aero-optical phenomena.

**Fig. 17:**
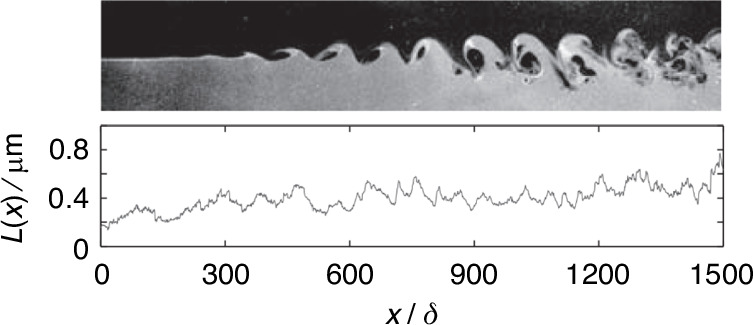
Two time-correlated flow visualization results and their optical path lengths of a supersonic mixing layer^65^

Based on the flow visualization results of supersonic mixing layer obtained by Zhao et al.^64^, Gao et al.^65^ computed the optical path lengths (*L*) of a supersonic mixing layer (as shown in Fig. 17), and analyzed the structure of the optical path length using wavelet methods. Additionally, Fassler et al.^66^ observed that the distortions in air–air case were mostly pressure-dominant, while helium–air case was predominantly mixing-dominant. Fassler et al.^58^ further investigated the effect of the mismatched temperature across the mixing layer created by blowing cool air over a flat window, proposing a new scaling method for aero-optical distortions in a temperature-mismatched, species-matched supersonic mixing layer, which exhibited an improved linear fit compared to the previous model. Castillo et al.^40^ investigated the aero-optical distortions arising from density fluctuations of the turbulent mixing layer over the optical window.

**Fig. 18:**
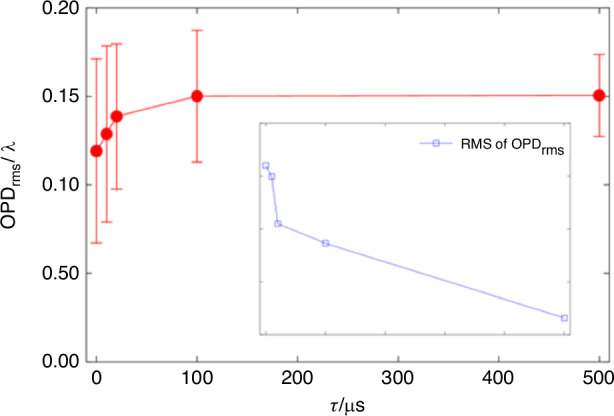
*OPD*_rms_ results under different exposure times^68^

After introducing typical flow structures, our attention will now shift to examining the aero-optical effects experienced by optical windows. In 1999, Pond et al.^6^ conducted a comprehensive analysis of the aerodynamic flow and its aero-optic effect for a side-mounted IR window. Their study aimed to quantify target image degradation, window heating and bending, and window structural failure probability due to aerothermal and aero-optic effects. The aero-optical effects induced by the complex flow structures around the hypersonic optical window are highly time-dependent, resulting in a significant difference in the imaging quality of the detector under different exposure times. In 1992, Kathman et al.^67^ conducted an experimental investigation to determine the variation in aero-optic effects as a function of camera exposure time. The data demonstrates a broad temporal transition from instantaneous image degradations to long-term image blur. Ding et al.^68^ observed that within the exposure time range of 6 ns~499 μs, as the exposure time increases, the corresponding *OPD*_rms_ of the high-order aberration gradually increases, while the magnitude of the increase gradually decreases, as shown in Fig. 18. As exposure time increases, the obtained high-order aberration wavefront stabilizes at a nearly constant value, which means that the imaging quality will not continue to deteriorate.

**Fig. 19:**
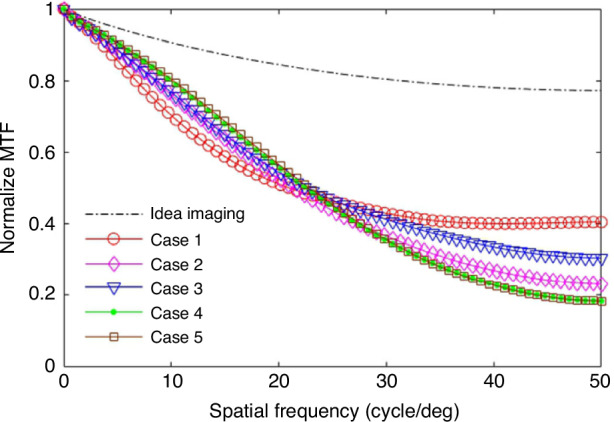
MTF distribution under different exposure times^68^

Further analysis indicates that the use of the optical transfer function (OTF) enables a more comprehensive analysis of the impact of exposure time on imaging quality. The amplitude component of the OTF (i.e., the modulus of the OTF) is called the modulation transfer function (MTF), which is commonly used to describe the contrast reduction of the image. Figure 19 shows the distribution of the MTF corresponding to the aero-optical transmission effects of the flow around the hypersonic optical window at different exposure times.

In Fig. 19, the horizontal axis, $v={({F}_{x}^{2}+{F}_{y}^{2})}^{1/2}$ represents the radial frequency distance in the two-dimensional spatial frequency domain, *F*_*x*_ and *F*_*y*_ denote the frequency parameters along the *x* and *y* directions in the Cartesian coordinate system, respectively. ${v}_{0}=\frac{{A}_{{\rm{D}}}}{\lambda f}$ signifies the cutoff frequency of the incoherent imaging system, *λ* is the wavelength of the light, and *f* is the focal length of the imaging system.

### Aero-optical transmission effects suppression

The suppression of aero-optical transmission effects fundamentally relies on regulating density fluctuations in the flow field. Castillo et al.^69^ conducted research on the wall-cooling effect on aero-optical distortions for the hypersonic boundary layer. With an increase in wall temperature, the density correlation length, away from the wall but inside the boundary layer, increases significantly for beam paths tilted in the downstream direction. Su^70^ performed an aero-optical analysis of a film-cooled optical window utilizing linear stability analysis. Three cases with different cooling gases are investigated, specifically, the commonly used air, a light gas (that is, helium), and a slightly heavier gas (that is, carbon dioxide). Among the three candidates, attributed to its low density, helium demonstrates superior performance in terms of *OPD*, which effectively minimizes density fluctuations within the flow, resulting in much better optical performance as compared to the other two gases.

The wall cooling method is another method that leverages the principles of the SRA or extended SRA (ESRA) principles to suppress the aero-optical effects. Specifically, this method involves reducing temperature to reduce the density fluctuation intensity. In 2010, Cress^71^ identified wall cooling as a potential method for mitigating aero-optic effects. Although implementing this method posed challenges in specific projects, the statistical model developed for the wall temperature effects on aero-optical wavefront aberrations predicted the existence of an optimal temperature that reduces *OPD*_rms_ by 80%. Smith and Gordeyev^72,73^ also employed wall cooling to suppress the aero-optical effects of the turbulent boundary layer. The total temperature of the boundary layer was reduced by using either all or part of the wall cooling method to reduce the intensity of the density fluctuation. Experimental results indicate that the *OPD*_rms_ was reduced by approximately 80% after cooling the development length of the entire boundary layer with this method^73^.

**Fig. 20:**
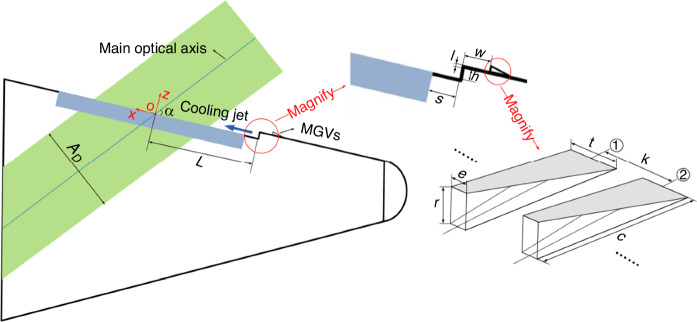
Schematic of testing model and flow control device (MVGs)^14^

**Fig. 21:**
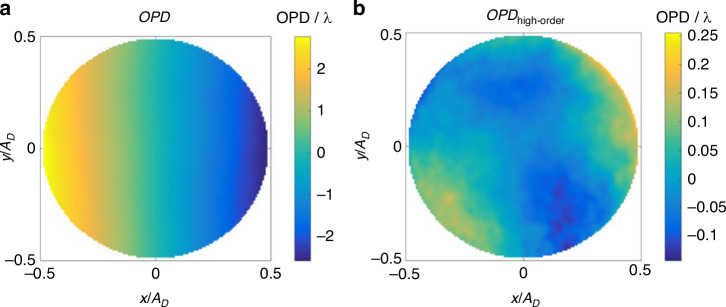
*OPD* results of a hypersonic optical window with film cooling^14^. **a** Result of total *OPD*. **b** Result of high-order component of total *OPD*

Prior discussions have highlighted the substantial influence of large-scale turbulence structures on the spatiotemporal distribution of density fluctuations to a significant extent. Theoretically, breaking down the large-scale eddy structures into smaller scales can reduce the intensity of density fluctuations, and consequently suppress the aero-optical transmission effects. Smith and Gordeyev^72^ designed a large eddy breakup (LEBU) device (resembling a thin wing) placed at a distance *h* = 0.5*δ* ~ 0.6*δ* from the wall in the TBL, suppressing the overall aero-optical effect by 33%. However, when this method was applied to a supersonic flow field, an additional wave structure could be introduced, requiring further evaluation. Freeman and Catrakis^74^ introduced plasma into the flow at various pulsing frequencies to achieve large-scale suppression (LSS) in turbulence. Reductions in the ensemble-averaged *OPD*_rms_ of up to 27% were achieved. As the dominant contributions to the aberrations in unforced flows are caused by large-scale organized structures, their findings indicate that the significant reductions in the forced experiments are induced by the large-scale suppression of the turbulent structures directly affected by the pulsed plasma actuator. Figure 20 shows the experimental model, which is a hypersonic optical window with a tangential supersonic gas film. Ding et al.^14^ arranged an array of MVGs at a distance of 20 mm upstream of the nozzle outlet, with a height of *r* = 1 mm (≈ 30%*δ*). The top view of a single MVG is a trapezoid with the top base *e* = 0.5*r*, the bottom base *t* = 1.5*r*, and the height *c* = 5*r*. The entire MVGs array includes 40 individual MVG, with a distance of 2 mm between adjacent vortex generators. ① and ② respectively represent the centerline (*z* = 0) of the corresponding MVG. Figure 21 are typical *OPD* results of a hypersonic optical window with film cooling)^14^.

**Fig. 22:**
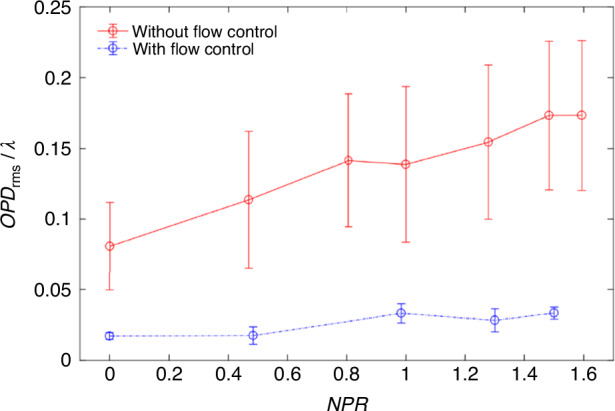
Relationship curve between *OPD*_rms_ and *NPR* with/without flow control^14^

**Fig. 23:**
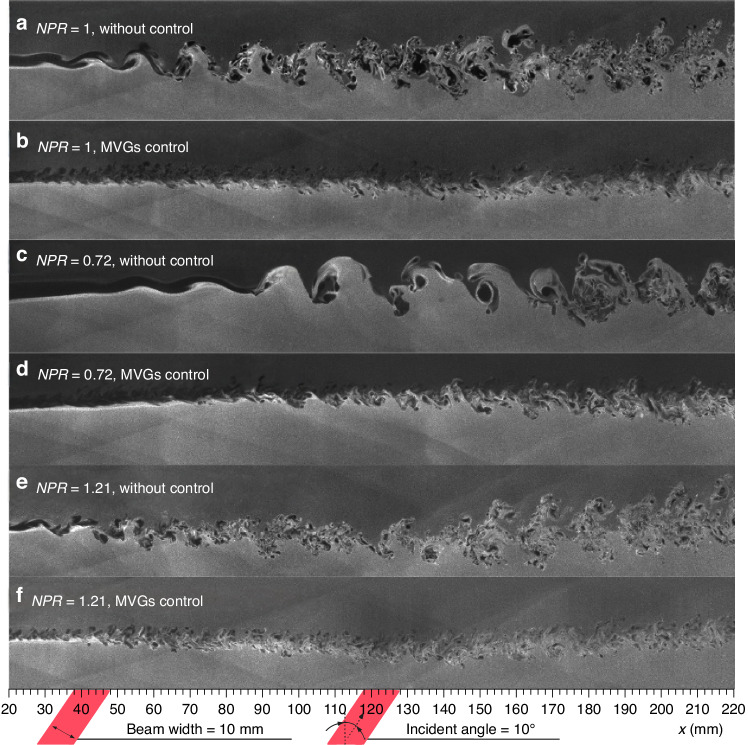
Comparison of flow visualization results for supersonic mixing layer before and after using the MVGs^75^. **a** NPLS result of supersonic mixing layer without control at *NPR* = 1. **b** NPLS result of supersonic mixing layer with MVGs control at *NPR* = 1. **c** NPLS result of supersonic mixing layer without control at *NPR* = 0.72. **d** NPLS result of supersonic mixing layer with MVGs control at *NPR* = 0.72. **e** NPLS result of supersonic mixing layer without control at *NPR* = 1.21. **f** NPLS result of supersonic mixing layer with MVGs control at *NPR* = 1.21

After removing the steady-lensing term and unsteady component from the *OPD*, the influence of *NPR* on *OPD*_high-order_ can be studied with and without flow control, with *OPD*_rms_ corresponding to *OPD*_high-order_ as the primary evaluation indicator. Based on *OPD*_rms_ results corresponding to *OPD*_high-order_ obtained under different conditions, the curve of *OPD*_rms_ versus *NPR* is plotted as shown in Fig. 22. The introduction of MVGs significantly suppressed *OPD*_high-order_ under different *NPR* conditions. In Fig. 22, “○“ and error bars represent the average and root mean square values of *OPD*_rms_ under the same condition, respectively. Additionally, the use of MVGs reduced the differences in *OPD*_high-order_ at different times, thereby improving the stability of the wavefront. In further, Ding et al.^75–77^ conducted systematic experimental and theoretical analysis research on the above-mentioned issue. They found that MVGs can effectively eliminate large-scale vortex structures in the supersonic mixing layer flow, suppress the mixing efficiency of the supersonic mixing layer, and reduce the thickness and density fluctuation of the supersonic mixing layer, as shown in Fig. 23. The analysis of *OPD*_rms_ results indicates that after MVGs control, the average, and standard deviation of *OPD*_rms_ decrease to different extents along the streamwise direction, leading to significant suppression of the aero-optical effects of the supersonic mixing layer.

Currently, there is a more stringent requirement for the suppression of aero-optical transmission effects in applications, such as airborne laser pointing and optical communication. Flow control methods, represented by MVGs can effectively reduce the generation of aero-optical distortions from the root cause. By coupling with end-stage aero-optical suppression methods, such as adaptive optics correction, it is theoretically possible to achieve superior suppression effects. In the future, we can attempt to find robust structured light with specific modes that maintain the entire structure of amplitude and phase unchanged when propagating through turbulent flow fields. In the past, optical angular momentum beams were considered as structured light with this potential due to their invariance of polarization components. In 2023, Forbes et al.^78^ from the University of the Witwatersrand proposed a new algorithm for finding robust structured light in atmospheric turbulence, which provides new insights for us to search for robust structured light in aero-optical transmission effects.

## Hypersonic aero-optical effect testing technology

The aero-optical wavefront induced by high-speed flow is characterized by a high degree of spatial non-uniformity (from the order of meters to the order of 10 μm)^15^ and temporal non-stationarity (up to MHz)^79–81^, and it demands that the measurement technology for aero-optical distortion wavefronts have higher spatial and temporal resolution. This requirement for high spatiotemporal resolution in aero-optical wavefront testing may be more stringent under hypersonic conditions. The current wavefront measurement technologies, notably Shack-Hartmann wavefront sensors (SHWFS), exhibit low spatiotemporal resolution that does not suffice for measuring aero-optical wavefront distortion under hypersonic conditions. Therefore, they are unable to support fundamental research on aero-optical effects and ground testing for hypersonic optical windows.

### High spatial-temporal resolution wavefront measurement technology

**Fig. 24:**
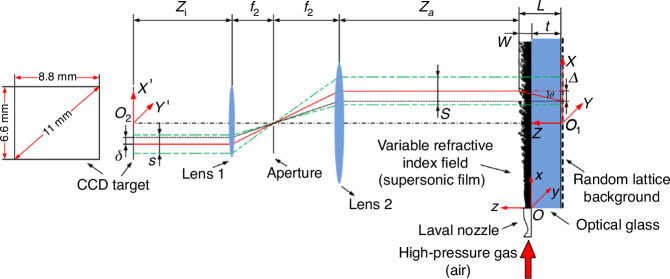
Schematic diagram of wavefront measurement based on near-field BOS technique under double telecentric configuration^84^

Figure 24 illustrates a schematic diagram of wavefront measurement utilizing the near-field background oriented schlieren (BOS) technique under a double telecentric configuration. In the double telecentric configuration, two focusing lenses are employed, with a circular aperture positioned at their shared focal point. Utilizing this far-field optical configuration for BOS measurements not only mitigates measurement errors associated with traditional techniques but also enhances the spatial resolution of the test^82,83^. By employing standard plano-convex lenses for the quantitative assessment of wavefront testing accuracy, the efficacy and precision of wavefront measurement based on near-field BOS technique under double telecentric configuration can be ensured. Under double telecentric configuration, various approaches for determining the spatial resolution, sensitivity, and dynamic measurement range of wavefront measurement based on near-field BOS technique have been suggested. Furthermore, investigations have been conducted to analyze the influence of the query window size and step size in cross-correlation calculations on the accuracy of wavefront testing^84^.

**Fig. 25:**
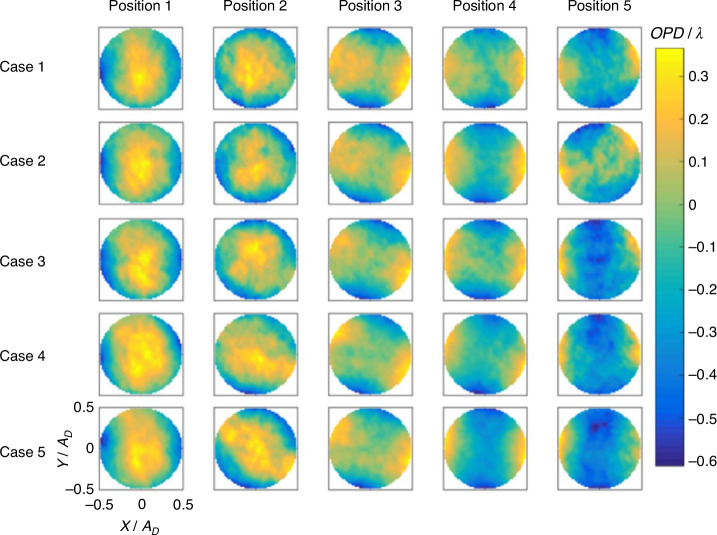
Transient wavefront results of supersonic film obtained by near-field BOS^84^

The step size of the query window should not be excessively small. Optimal results are often obtained when it is set to half of the query window size, leading to relatively ideal reconstruction accuracy. However, this also depends on the information of the observed aberration field. For SHWFS, the spacing between the centers of the microlenses on its microlens array cannot be smaller than the microlens diameter. Due to limitations in the performance indicators related to dynamic range, it is not feasible to design the microlens size infinitely small. This limitation, to a certain extent, constrains the enhancement of spatial resolution in wavefront testing using SHWFS. In general, BOS technique utilizes backlit panels or LED light sources, which are limited by light intensity and cannot measure instantaneous wavefront with extremely short exposure times. By employing an eight-cavity Nd: YAG laser along with a framing camera, featuring a laser single pulse energy 300 mJ and a pulse width 6 ns, we are able to achieve instantaneous (6 ns) aero-optical wavefront measurement based on BOS. Additionally, the minimum time interval can reach 0.2 μs. When employing the wavefront measurement based on BOS, via reducing the query window step size, it is possible to increase the number of equivalent sub-apertures times within the optical observation aperture without sacrificing the dynamic range of wavefront testing. Figure 25 illustrates the transient wavefront results of supersonic gas film at various cases and positions obtained using this technique. The actual spatial resolution used is 111 × 111, and the maximum spatial resolution can reach 447 × 447. Compared to the highest spatial resolution (47 × 35) of SHWFS in the WFS20 series produced by Thorlabs, the spatial resolution has been increased by at least 120 times^84^.

**Fig. 26:**
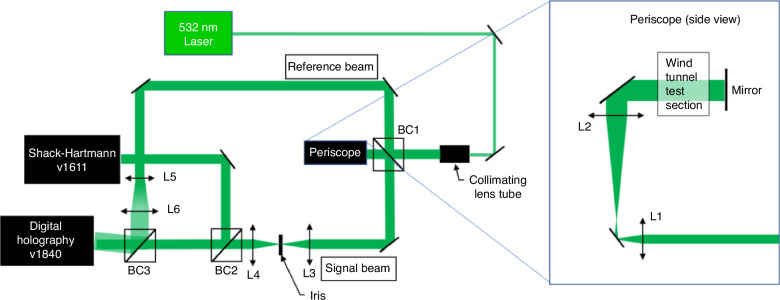
Optical system layout in a z-configuration for Schlieren imaging and wavefront sensing^87^

Digital holography wavefront sensor (DHWFS) offers superior spatial and temporal resolution compared to SHWFS, making it increasingly popular in supersonic/hypersonic research over the past decade. In 2015, Spencer et al.^85^ utilized DHWFS to measure wavefront distortion caused by atmospheric turbulence. In 2019, Wilcox et al.^86^ employed DHWFS to measure wavefront distortion induced by minor shocks present in the test section of a supersonic wind tunnel. In 2023, Chu et al.^87^ from the University of Notre Dame used DHWFS in conjunction with SHWFS to simultaneously measure wavefront distortion in a local supersonic region overlying a two-dimensional partial cylinder within a supersonic wind tunnel, as shown in Fig. 26. The findings revealed that DHWFS effectively addressed the limitations of SHWFS in measuring large wavefront gradients near shockwaves^87^.

**Fig. 27:**
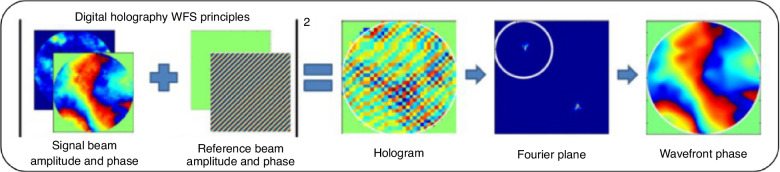
Digital Holography WFS operational schematic^88^

DHWFS is based on the principles of holography, a method for recording and reconstructing the wavefront of light. A signal beam of light from the object of interest interferes with a known reference wave. The interference pattern contains both the amplitude and phase information of the object wave, essentially creating a hologram of the wavefront. A digital sensor, typically a CCD (charge coupled device) or CMOS (complementary metal-oxide-semiconductor) camera, is used to record the interference pattern. The recorded hologram is a 2D intensity image that encodes the 3D information of the object wavefront. Using Fourier analysis, the phase information can be reconstructed, providing the full complex field of the signal wave. This process is depicted in Fig. 27^88^.

Compared to BOS technique, DHWFS is more sensitive to wavefront distortion. Its direct measurement of the integrated *OPD* along the propagation path enables the measurement of large wavefront gradients near the shock wave. However, its optical path is complex, and it involves Fourier transformation to extract the Fourier domain to filter out interference fringes, which hampers the extraction of fine structures, including minute, strongly compressed features, thereby limiting the maximum resolution. In contrast, BOS features a straightforward optical path, variable spatial resolution, and sub-pixel displacement recognition. Its disadvantage is that it is less sensitive to wavefront distortion.

### Synchronous measurement of aero-optical distortion information and flow information

Essentially, the aero-optical wavefronts are primarily determined by the density distribution characteristics of the flow field. Therefore, the spatiotemporal features of the aero-optical distortion wavefronts are closely related to the corresponding spatiotemporal characteristics of flow density. Simultaneous measurement of aero-optical distortion wavefront information and flow information can effectively support the investigation of the underlying flow mechanisms responsible for the spatiotemporal characteristics of aero-optical distortion wavefronts^89^. This approach provides insight into the fundamental understanding of the mechanisms that drive the generation of aero-optical distortion from the perspective of flow, and thus, offers support for suppressing the generation of aero-optical effects at their source.

**Fig. 28:**
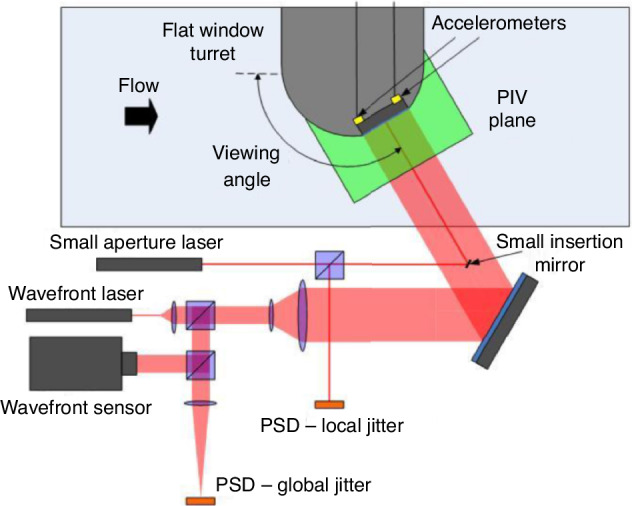
The schematic of the experimental setup^91^

In 1996, Gordon et al.^90^ performed a high-frequency crossed-beam correlation experiment to investigate the mean-squared fluctuating density, convection speed, and characteristic turbulent coherence length of a supersonic turbulent mixing layer. The motion of the beam was detected by two quadrant detectors, and the output signals were recorded after being digitally sampled at a rate of 5 MHz. In 2012, a clever experiment was designed by Lucca et al.^91^ from the University of Notre Dame to synchronously measure local jitter, 2D wavefronts, and accelerometer measurements in the flow over a flat window turn, as shown in Fig. 28. And successfully separated the mechanically related component of the jitter from the aero-optical component using a linear stochastic estimation technique. At the same time, they also attempted to introduce PIV technique to synchronously measure the flow field while measuring the aero-optical jitter, in order to address important questions about the origin and dynamics of the station aero-optical structure by analyzing the flow itself^91^.

**Fig. 29:**
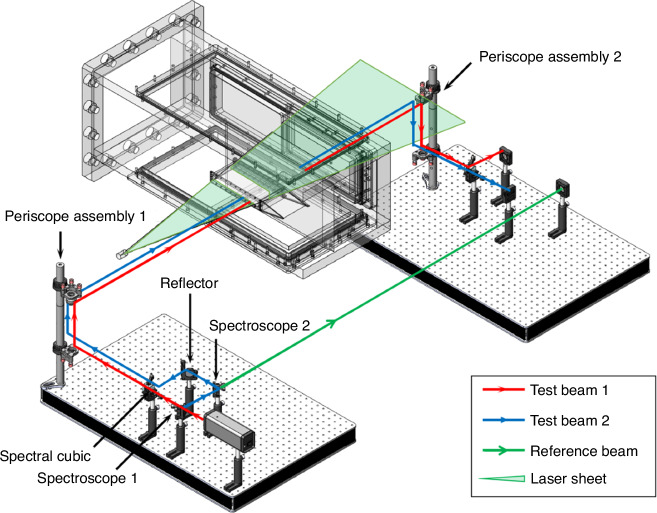
Schematic of NPLS and MP synchronous measurement system

**Fig. 30:**
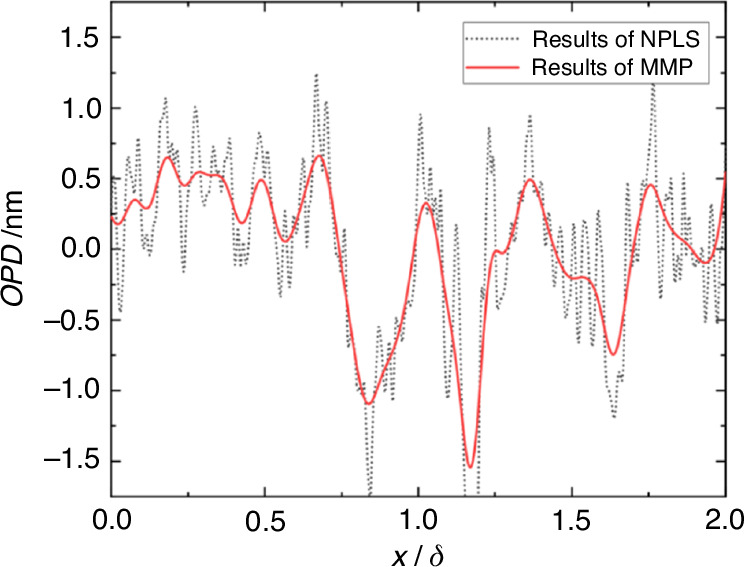
NPLS and MP synchronous wavefront measurement results

Inspired by the work of Lucca et al., we attempted to build a synchronous measurement system, as shown in the Fig. 29. MP technique can achieve high-frequency continuous acquisition of one-dimensional wavefronts. The wavefront testing technique based on Nano-tracer-based Planar Laser Scattering (NPLS) technique can achieve one-dimensional wavefront data while displaying the flow^92^. By combining MP technique with NPLS technique, a synchronous measurement system for optical distortion and visualizing flow field had been established. This platform enabled the synchronous measurement of the instantaneous density field of supersonic turbulent boundary layers and their one-dimensional aero-optical distortion wavefronts. The synchronous test system, as illustrated in Fig. 29, includes the NPLS system’s laser sheet and the MP test beam group being set on the same horizontal plane. Based on the Taylor frozen hypothesis, NPLS and MP techniques can be used to measure the one-dimensional wavefront test results of the same “instantaneous” flow field. The wavefront results obtained from the two technologies are illustrated in Fig. 30.

### Testing system for aero-optical wavefront of hypersonic optical window

**Fig. 31:**
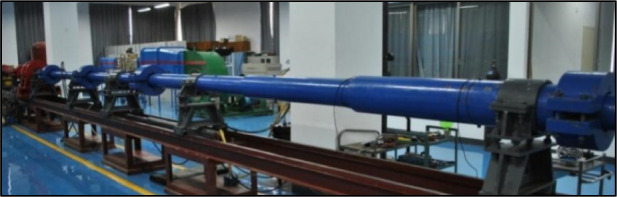
KD-01 hypersonic gun wind tunnel^93^

The KD-01 hypersonic gun tunnel at the Hypersonic Aero-optical Effects Laboratory (HAOEL) of the National University of Defense Technology, as shown in Fig. 31, is composed of a driving section, a driven section, an experimental chamber, and a vacuum tank. The total length of the wind tunnel is 42 m, with inner diameters of 103 mm for the driving and driven sections, and an exit diameter of 500 mm for the nozzle. The wind tunnel employs a light-piston driving method, with an effective operation time of approximately 25 ms^93^.

**Fig. 32:**
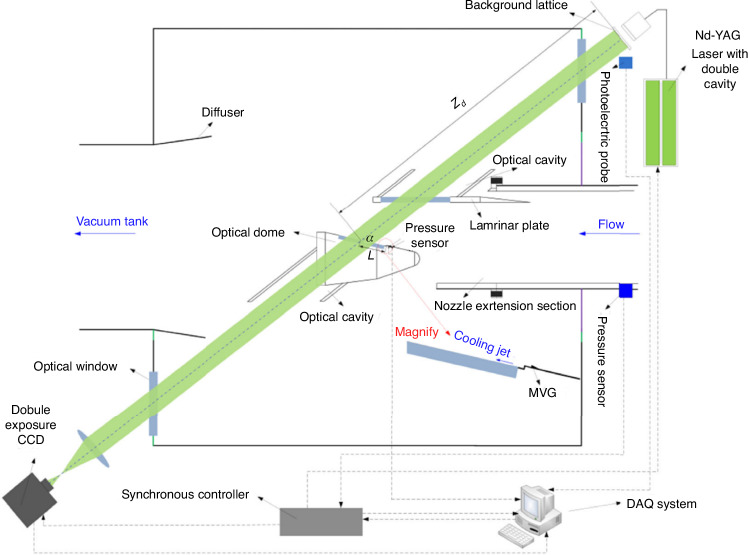
Schematic diagram of ground test device for hypersonic aero-optical effects^68^

As shown in Fig. 32, the influence of the boundary layer from the axisymmetric nozzle and the rhombic zone wave system on the aero-optical wavefront testing was eliminated by appropriately extending the wind tunnel nozzle and installing a laminar flow plate. Optical cavities were installed at the top of the laminar flow plate and the bottom of the optical window to eliminate the effects of the flow and jet boundaries. An optical window installed on the experimental chamber meets the requirements for frontal imaging of the hypersonic optical window. With the principles of wavefront measurement based on BOS, the wavefront testing system for the hypersonic optical window was established, as shown in Fig. 32.

**Fig. 33:**
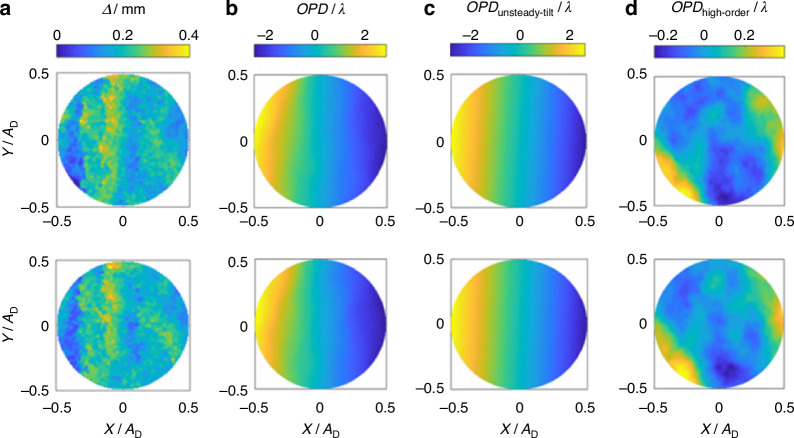
Transient displacement and its corresponding *OPD* of hypersonic optical window^68^. **a** Transient displacement result of hypersonic optical window. **b** Transient *OPD* result of hypersonic optical window. **c** Unsteady-tilt component of *OPD*. **d** High-order component of *OPD*

To enable to achieve short exposure (transient) wavefront measurements, a dual-cavity Nd-YAG laser (with a wavelength of *λ* = 532 nm, pulse width of 6 ns, maximum single pulse energy of up to 500 mJ, actual usage of 200 mJ, an inter-frame time Δ*t* = 5 μs) was used to illuminate a pre-designed random background array. The camera used for the transient test is a double exposure camera, featuring a CCD pixel linear size of *l*_p_ = 5.50 μm, and a standard resolution of up to 6576 × 4384 pixels. The laser and the camera are operated via a synchronization controller with a control accuracy of up to 250 ps, ensuring that the CCD is exposed in synchronization with the laser illumination of the background. The above hardware parameters determine the exposure time *τ* = 6 ns for the transient wavefront test. Figure 33 shows the displacement *Δ* cloud image acquired utilizing the transient wavefront measurement system (Fig. 32) with an inter-frame time of 5 μs. Since the displacement data reflects the deflection of the light by the flow field, it is essentially equivalent to the surface gradient of the wavefront. Based on this gradient value, wavefront reconstruction is accomplished through the Southwell method, and the obtained *OPD* result is shown in Fig. 33.

## Summary and discussion

The confluence of thermal effects and optical transmission effects in hypersonic aero-optical effects present significant challenges to optical imaging detection of aircraft. Based on the supersonic film cooling, the aero-optical thermal effects encountered by the hypersonic optical window can be effectively alleviated. Nevertheless, this strategy inevitably engenders more complex flow structures near the window, consequently intensifying aero-optical transmission effects. This paper reviews and summarizes recent research advancements, pertaining to the aforementioned issue, with the objective of furnishing guidance and support for researchers engaged in this domain.

With the ongoing escalation in-flight Mach numbers and the expansion of airspace, the airflow surrounding aircraft exhibits characteristics with high Mach numbers, high enthalpy, and high Reynolds numbers. Such conditions pose increasingly formidable challenges for optical imaging and detection within hypersonic optical windows. To tackle the challenges outlined above, in the theoretical foundation of hypersonic aero-optical effects, the induced mechanisms of aero-optical effects in high enthalpy turbulent flow fields at hypersonic speeds should be focused. It is essential to elucidate the influence of gas self-radiation on the quality of optical imaging. The fundamental theory of aero-optical effects under real gas conditions should be refined in further. In the realm of hypersonic aero-optical testing technology, there exists a requirement for synchronous measurement of high-speed and high-resolution distortion wavefronts. Such measurements can furnish insights into the evolution of hypersonic flow and the associated aero-optical distortions. To address this need, a novel principle of distortion wavefront testing is currently under development, aiming to achieve both high-spatial resolution and high-temporal resolution. This innovation will serve as the technical foundation for investigating hypersonic aero-optical effects. In the domain of hypersonic aero-optical suppression, there should be ongoing exploration into methodologies that integrate flow control with optical correction to effectively mitigate aero-optical effects. Models of aero-optical effects are currently being developed based on the integration of flow parameters and optical parameters to enhance the efficacy of hypersonic aero-optical effects suppression.

## Data Availability

The data that support the plots within this paper and another finding of this study are available from the corresponding author upon reasonable request.
